# Supplementary material for: Integrating multiplexed imaging and multiscale modeling identifies tumor phenotype conversion as a critical component of therapeutic T cell efficacy
Source: Cell Syst. 2024 Apr 17;15(4):322–338.e5. doi: 10.1016/j.cels.2024.03.004 (PMC11030795; doi:10.1016/j.cels.2024.03.004)
Supplement: Document S1. Figures S1–S4, Table S1, and supplemental references [file mmc1.pdf]

Cell Systems, Volume *15*

Supplemental information

Integrating multiplexed imaging and multiscale modeling identifies tumor phenotype conversion as a critical component of therapeutic T cell efficacy

John W. Hickey, Eran Agmon, Nina Horowitz, Tze-Kai Tan, Matthew Lamore, John B. Sunwoo, Markus W. Covert, and Garry P. Nolan

SUPPLEMENTAL FIGURES 1-9

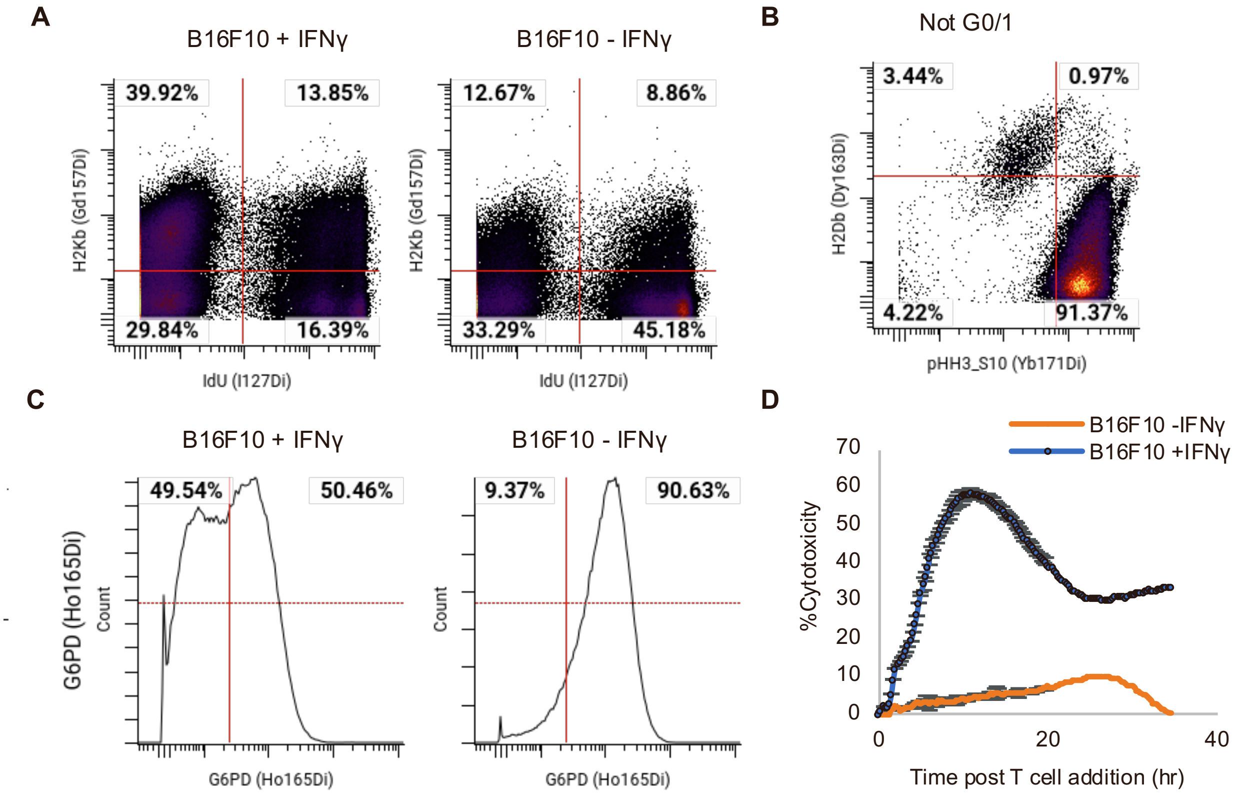

Supplemental Figure 1: Treatment of tumor cells with IFNγ causes profound metabolic and inflammatory molecular processes that influence T cell killing. **A-C)** CyTOF staining of B16F10 tumor cells either cultured with or without the presence of IFNγ. **A)** IdU and H2Kb staining shown for all cells. **B)** pHH3-S10 and H2Db staining shown for cells positive for IdU. **C)** G6PD staining shown for all cells. **D)** Percent killing of cognate tumor cells over time by expanded therapeutic T cells pre-incubated with IFNγ or not. Tumor and T cells were incubated at a 1:1 ratio (mean of n=3 replicates with error bars showing SEM), is an extended timeline of Figure 2C.

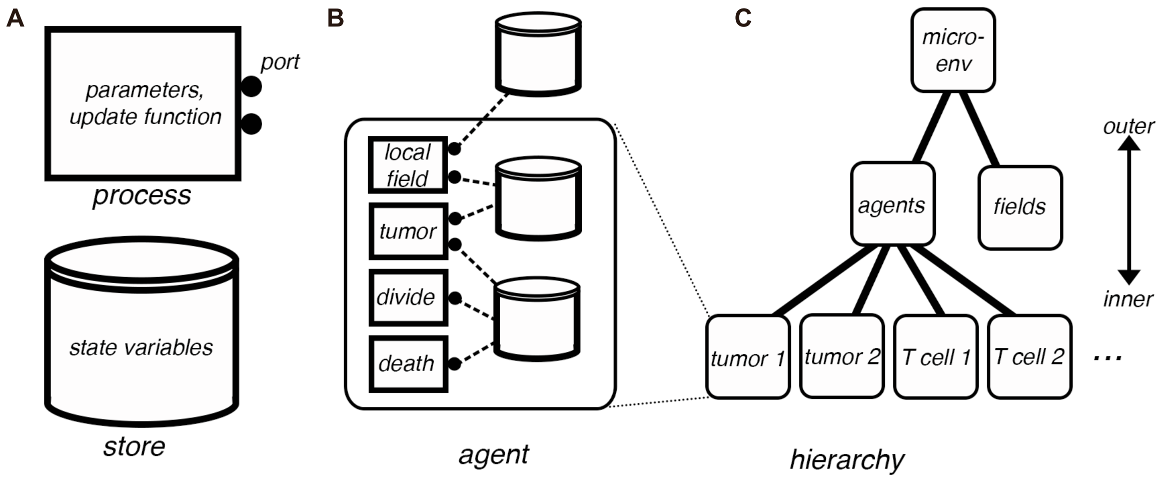

Supplemental Figure 2: Schematic of the tumor/T cell microenvironment model's Vivarium interface, illustrating the simulation's formal structure. **A)** *Vivarium's* basic elements. The *process*, shown as a rectangular flowchart symbol, is a modular model that contains the parameters, an update function, and ports. The *store*, shown as the flowchart symbol for a database, holds the state variables and schemas that determine how updates are handled. **B)** *Agents* are bundles of *processes* and *stores* wired together (called *composites* in *Vivarium* terminology) across a single level. The figure shows the main tumor process wired with processes called local field (for reading the external concentrations), divide (for dividing the cell), and death (for removing the cell from the simulation). **C)** Cell agents are *compartments* embedded in a hierarchy depicted here as a hierarchical network with discrete layers. Outer *compartments* are shown above and inner compartments below. The cells all exist under "agents" and the external fields are held in an adjacent *store* called "fields".

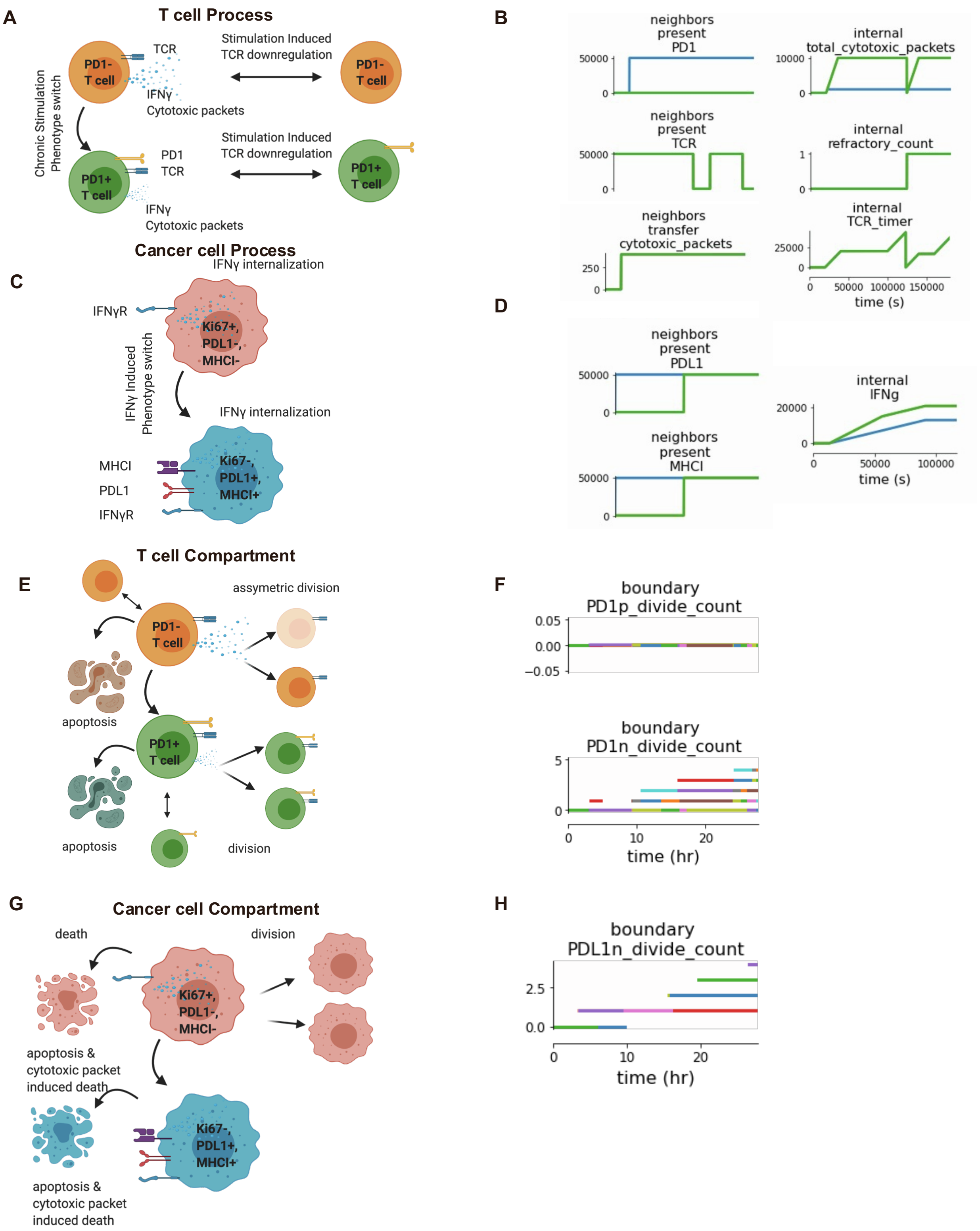

Supplemental Figure 3: Representation and development of individual components of the multiscale agent-based model. **A)** T cell process is composed of two T cell states. PD-1$^-$ CD8$^+$ T cells become PD-1$^+$ T cells upon chronic stimulation and both PD-1$^+$ T cells and PD-1$^-$ T cells can downregulate TCR. Both T cell types express TCR, IFNγ, and produce cytotoxic packets. Molecular regulation is governed by activation and stimulation of tumor cells. **B)** Representative

output of simulating only the T cell process. Each graph represents a specific molecule or molecular process that is being tracked within the simulation, and the first word describes the level in which that molecular process is connected to other pieces of the simulation. Starting with top-to-bottom and right-to-left, *neighbors present PD-1* measures the surface level ligand expression of the molecule PD-1 on a T cell; *internal total cytotoxic packets* measures the total number of cytotoxic granules within a T cells over time; *neighbors present TCR* measures the surface level ligand expression of the T cell receptor on a T cell; *internal refractory count* measures the total number of times T cells have been activated and entered a refractory state post stimulation; *neighbors transfer cytotoxic packets* measures the total number of cytotoxic granules a T cells is outputting to tumor cells over time; *internal TCR timer* measures the total time that T cells have been activated for. **C)** Tumor cell process is composed of two tumor cell states. Ki67^{+} PDL1^{-} MHCI^{-} tumor cells can become Ki67^{-} PDL1, MHCI^{+} tumor cells upon exposure to IFNγ. Both tumor cell types express IFNγR. Molecular regulation is governed by interaction with T cells. **D)** Representative output of simulating only the tumor cell process. Starting with top-to-bottom and right-to-left, *neighbors present PDL1* measures the surface level ligand expression of the molecule PDL1 on a tumor cell; *internal IFNg* measures the total number of IFNγ molecules a tumor cell has taken up over time; *neighbors present MHCI* measures the surface level ligand expression of MHC-I on a tumor cell. **E)** The T cell compartment extends the T cell process by adding division and death processes that can be asymmetric. **F)** Representative output of simulating the T cell compartment. Starting with top-to-bottom, *boundary PD-1p divide count* measures the number of times the PD-1^{+} CD8^{+} T cells have divided in the course of the simulation; *boundary PD-1n divide count* measures the number of times the PD-1^{-} CD8^{+} T cells have divided in the course of the simulation. **G)** The tumor cell compartment adds proliferation and death processes. **H)** Representative output of simulating the tumor cell compartment, such as *boundary PDL1n divide count* measures the number of times the PDL1^{-} tumor cells have divided in the course of the simulation.

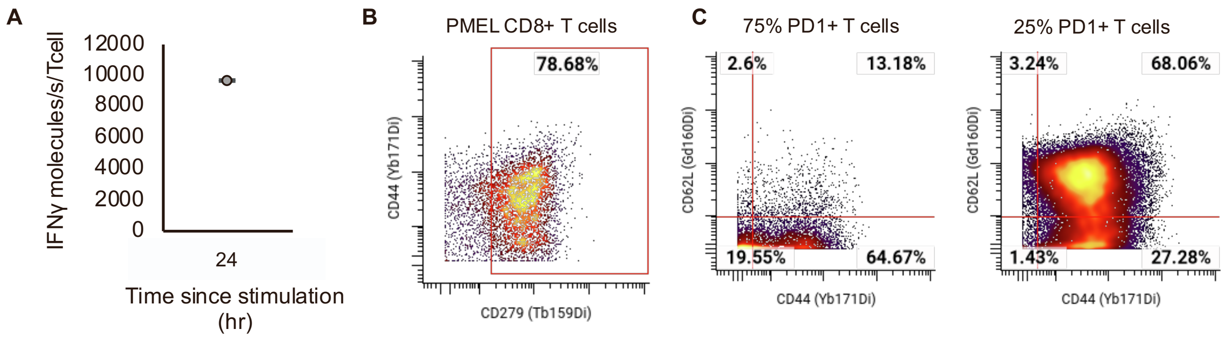

Supplemental Figure 4: Conditions used to initialize the T cell phenotype in the multiscale model. **A)** Average amount of IFNγ produced by T cells after restimulation post-10 day culture period as measured by an ELISA assay (n=2 technical replicates). **B)** Nearly 80% of restimulated T cells used for the *in vitro* killing assay express PD-1 (CD279) as plotted versus CD44 as measured by CyTOF. This condition was used to initialize the T cell phenotype for the agent-based model. **C)** Percent of memory versus effector cells determined post stimulation by CyTOF for both the 75% PD-1+ T cell condition and the 25% PD-1+ T cells condition (stimulated in presence of 2HC).

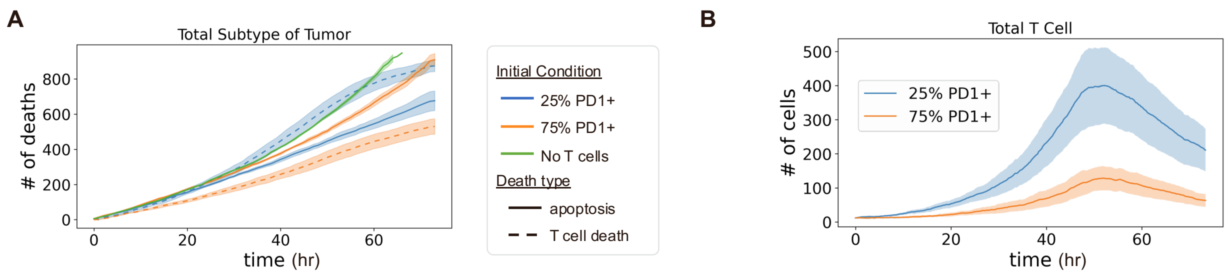

Supplemental Figure 5: Agent-based modeling results of treatment with 25% PD-1$^+$ T cells, 75% PD-1$^+$ T cells, or no T cells. **A)** Total number of tumor cell deaths quantified for T cell-induced killing and apoptotic events over 60-h simulation of treatment with 25% PD-1$^+$ T cells, 75% PD-1$^+$ T cells, or no T cells (mean of n=4 replicates with shading showing SEM). **B)** Number of T cells during 60-h simulation of treatment with 25% PD-1$^+$ and 75% PD-1$^+$ T cells (mean of n=4 replicates with shading showing SEM).

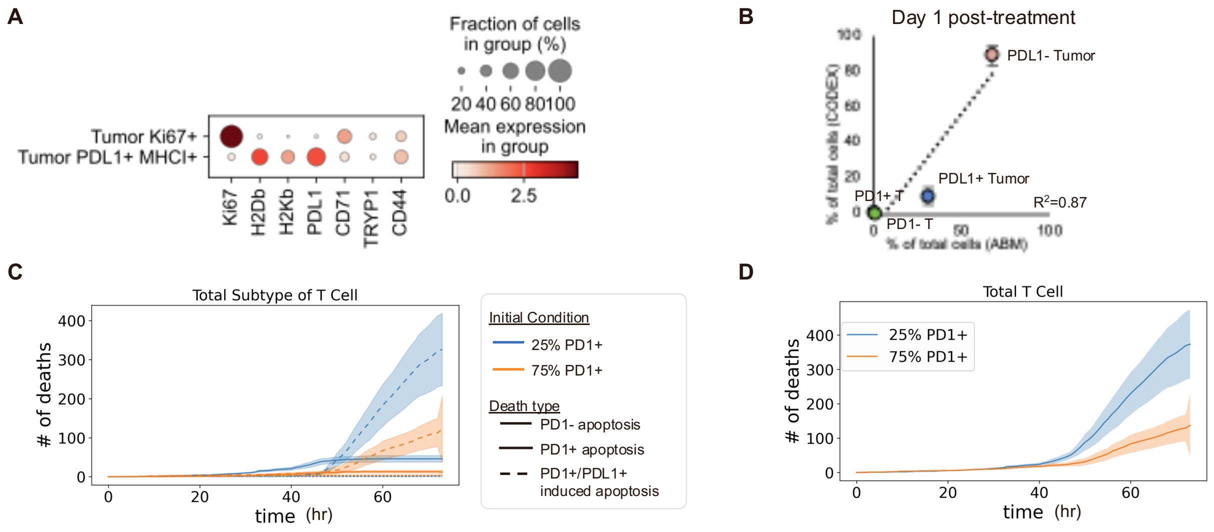

Supplemental Figure 6: Therapeutic T cells cause tumor phenotype conversion which is dependent on initial T cell phenotype. **A)** Average target molecule expression in either Tumor Ki67^{+} or Tumor PDL1^{+} MHCI^{+} cells as measured by CODEX multiplexed imaging following treatment with T cells (n=17 tumors, and >1 million cells). **B)** Correlation plots of percent of PD-1^{+} CD8^{+} T cells (green), PD-1^{-} CD8^{+} T cells (orange), PDL1^{-} Tumor (salmon), and PDL1^{+} Tumor (blue) cells in tumor samples after 3 days of T cell therapy for CODEX multiplexed imaging of *in vivo* experiments versus *in silico* simulations (n=4 replicates per group). **C)** Total number of T cell deaths grouped by type in tumors treated with 25% PD-1^{+} T cells or 75% PD-1^{+} T cells (mean of n=4 replicates with shading showing SEM). **D)** Number of total T cell deaths in tumors treated with 25% PD-1^{+} T cells or 75% PD-1^{+} T cells (mean of n=4 replicates with shading showing SEM).

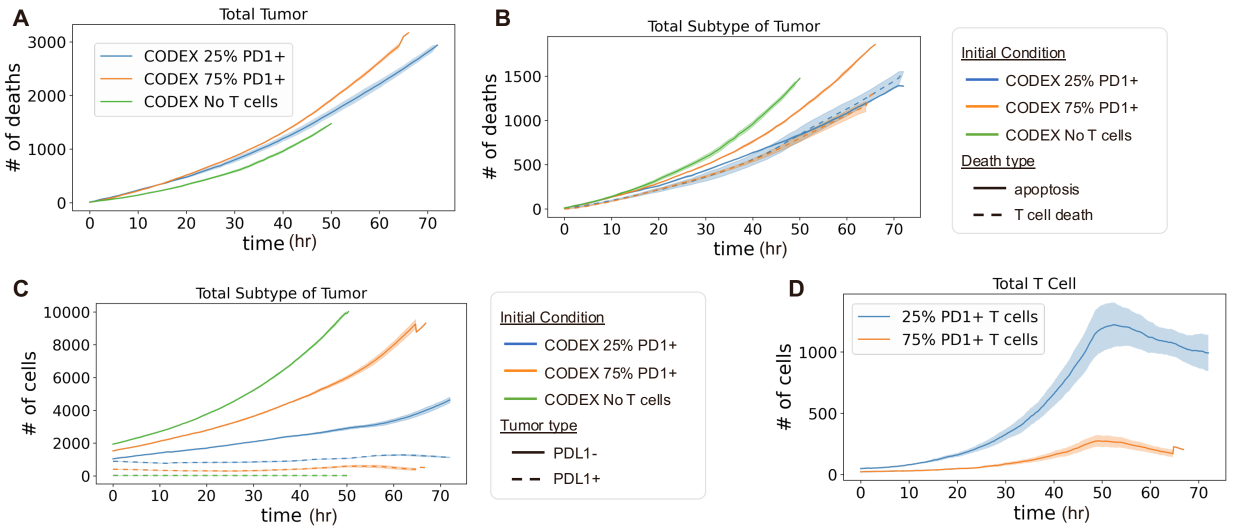

Supplemental Figure 7: Results from agent-based modeling simulations initialized with CODEX multiplexed imaging data. **A)** Total number of tumor cell deaths in simulations of treatment with 25% PD-1$^+$ T cells, 75% PD-1$^+$ T cells, or no T cells. B) Tumor cell deaths due to T cell-induced killing and apoptotic events in simulations of indicated treatments. **C)** Numbers of PDL1$^+$ and PDL1$^-$ tumor cells for each of the three treatment groups across the simulation. **D)** Number of T cells across the duration of the agent-based model for 25% PD-1$^+$ and 75% PD-1$^+$ T cell treatment conditions. For all panels: mean of n=4 replicates with shading showing SEM.

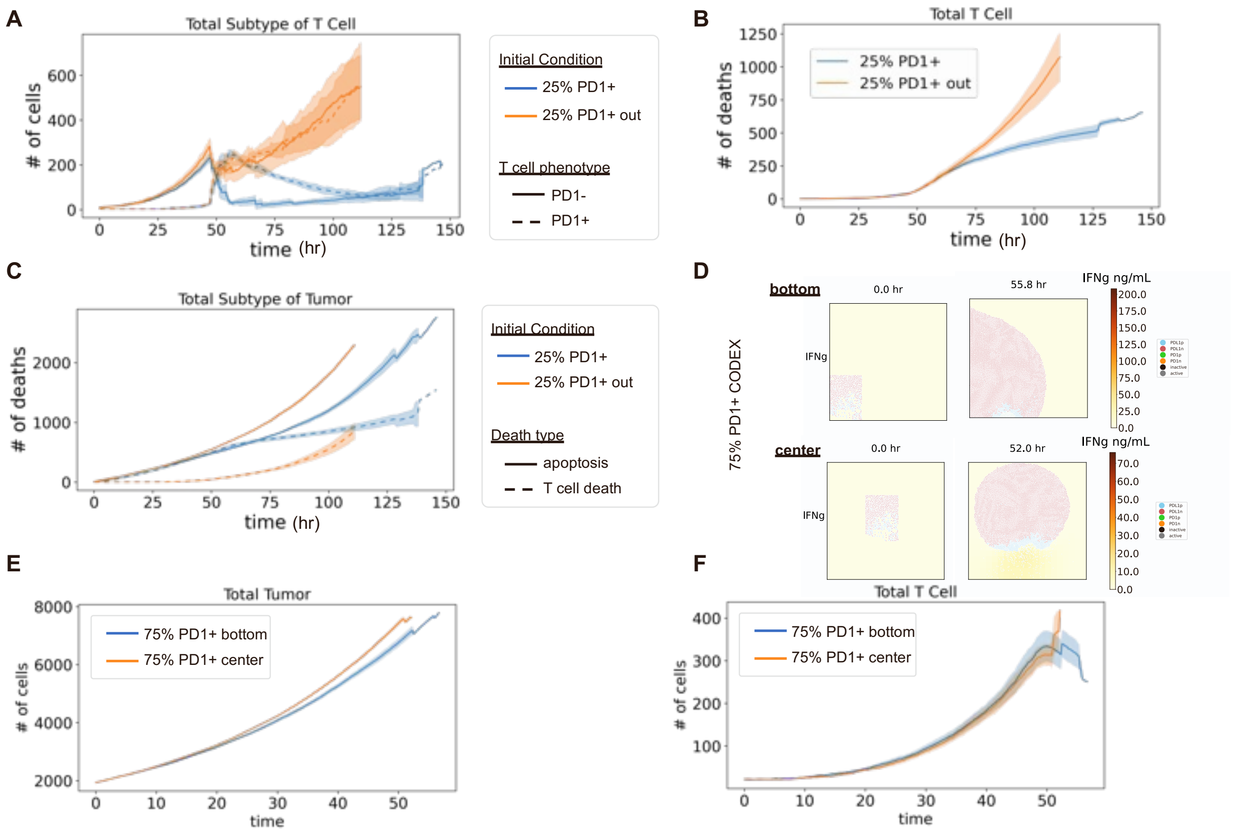

Supplemental Figure 8: Initialization of therapeutic T cells outside the tumor bed allows T cell phenotype preservation and greater numbers of T cells but faster tumor growth. **A)** Number of T cells by phenotype (PD-1^{+} and PD-1^{-}) quantified across the duration of the agent-based model for 25% PD-1^{+} initialized outside and 25% PD-1^{+} T cell initialized inside conditions. **B)** Total number of T cell deaths quantified for 25% PD-1^{+} initialized outside and 25% PD-1^{+} T cell initialized inside conditions. **C)** Total number of tumor cells deaths by T cell-induced killing and apoptotic events by tumor cell phenotypes in simulations with 25% PD-1^{+} initialized outside and 25% PD-1^{+} T cell initialized inside conditions. In all panels: mean of n=4 replicates with shading showing SEM. **D-F)** Different initializations of location of cells within the modeling frame for 75% PD-1^{+} T cell CODEX multiplexed data, with **D)** snapshots of initial and later timepoints in the modeling, **E)** Number of tumor cells and **F)** number of T cells compared between the same treatment group started in the bottom or center as a function of simulation time (mean of n=5 replicates with shading showing SEM).

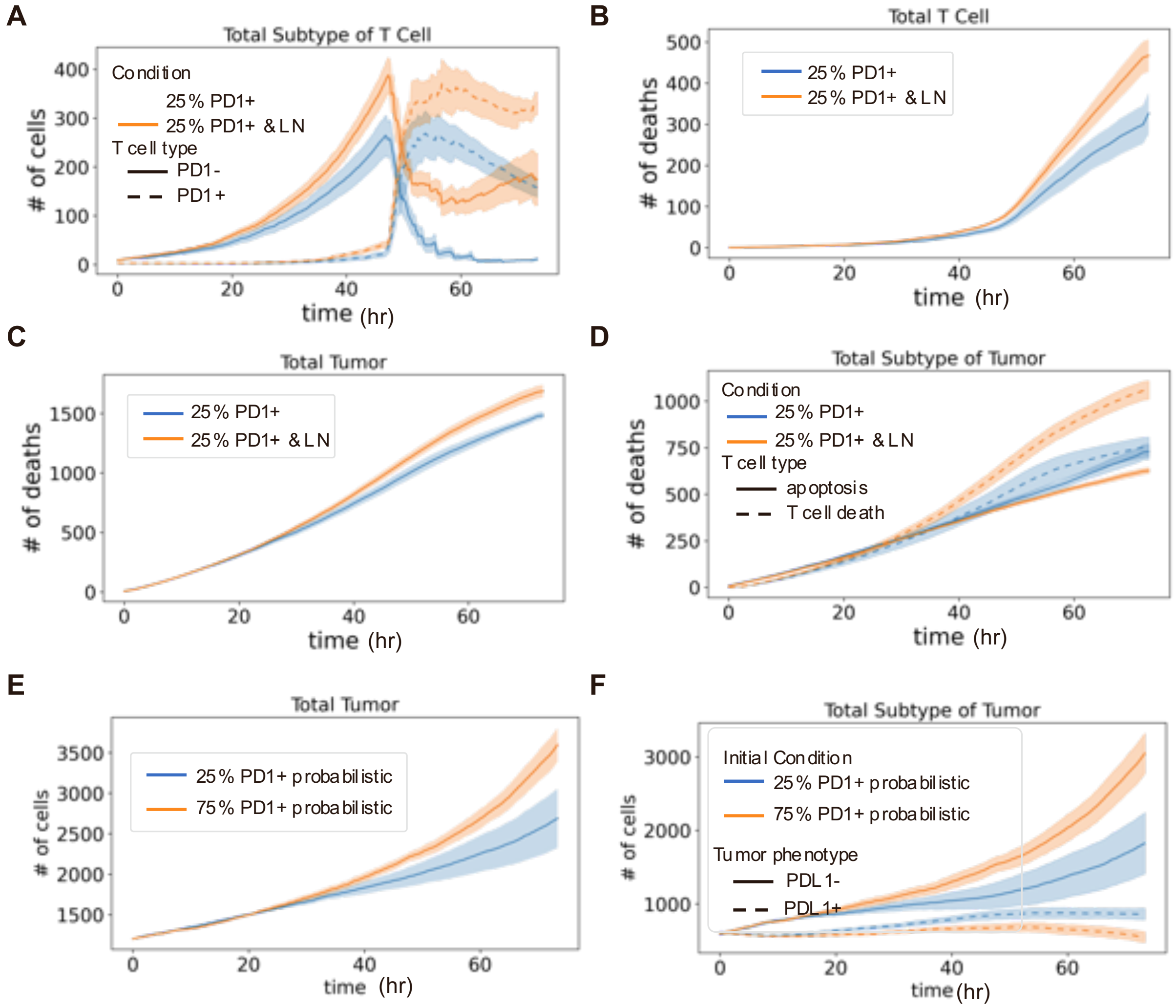

Supplemental Figure 9: Adding dendritic cells and lymph nodes to the tumor and T cell simulations enables greater numbers of sustained T cells over time and better control of tumor growth. **A-D)** Comparing simulations with added dendritic cells and LN processes versus simulations without LN and dendritic cells for: **A)** Number of T cells separated by phenotype, **B)** Total number of T cells within the tumor microenvironment over time quantified for 25% PD-1^+ with and without lymph node (and dendritic cell) processes. **C)** Total number of T cell deaths quantified for 25% PD-1^+ with and without lymph node (and dendritic cell) processes. **D)** Total number of tumor cells deaths by T cell-induced killing and apoptotic events by tumor cell phenotypes in simulations with 25% PD-1^+ with and without lymph node (and dendritic cell) processes. In all panels A-D: mean of n=8 replicates with shading showing SEM. **E-F**) Comparing simulations of tumors treated with 25% PD-1^+ T cells or 75% PD-1^+ T cells simulated with a probabilistic T-cell activation and refractory timing mechanism and showing **E)** Total number of tumor cells and **F)** total number of subtype of the tumor cells over simulation time (n=3-5 replicates with shading showing SEM).

SUPPLEMENTAL TABLES

Supplemental Table 1: Key model parameters and the source from the literature.

Model name	Value	Source	Location	Rationale/Explanation
bounds	Vary	Multiplexed Imaging Data	main.py	Size of tumor
Depth	15 μm			Based on an average single cell diameter
n_tumors	Vary			Number of tumors found
n_tcells	Vary			Number of T cells found
n_dendritic	Vary			Number of T cells found
dendritic_state_active	Vary			Number of activated dendritic cells found
diameter	7.5 μm	Multiplexed Imaging Data	t_cell.py	Diameter of average T cell
initial_PD-1n, cell_state	Vary	Multiplexed Imaging Data, CyTOF data		Based on percentage of cells that are PD-1- when transferred in
Location [x, y]	Vary	Multiplexed Imaging Data		Spatial position of T cells in simulation
PD-1	Vary	Multiplexed Imaging Data		Total protein expressed of PD-1
IFNg	Vary	CyTOF Data		Number of IFNg that are produced per T cell upon activation
PDL1_critical_number, ligand_threshold	1e4 (count/cell)	[1]		Threshold for neighboring cell to engage PD-1 on T cells
TCR_downregulated	0			Number of TCR that are on a T cell post downregulation
TCR_upregulated	50000 (count/cell)			Number of TCR that are on a T cell
refractory_count_threshold	3 (assuming stimulated 2 during in vitro activation)			Number of times that T cells become stimulated before becoming exhausted
PD-1n_divide_threshold	5			Number of times that T cells divide before becoming exhausted
activation_time	21600 s	[2,3]		Activation enables 6 hours of activation and production of cytokines before enters refractory state
activation_refractory_time	43200 s			refractory period of 18 hours (plus original 6

				h activation time counted) after activation period with limited ability to produce cytokines and cytotoxic packets and to interact with MHCI
death_PD-1p_14hr	0.35 (within 50400 s)	[4]		Likelihood of T cell death for PD-1+ T cell
death_PD-1n_14hr	0.1 (within 50400 s)			Likelihood of T cell death for PD-1- T cell
death_PD-1p_next_to_PDL1p_14hr	0.475 (within 50400 s)	[5,6]		Likelihood of T cell death for PD-1+ T cell when engaging with PDL1+ cell
PD-1n_IFNg_production	1.62e4 molecules/cell/s	[7,8]		Number of molecules of IFNg a PD-1- T cell produces
PD-1p_IFNg_production	1.62e3 molecules/cell/s			Number of molecules of IFNg a PD-1+ T cell produces
PD-1n_growth_28hr	0.9 (within 100800 s)	[4,9]		Likelihood of T cell division for PD-1- T cell
PD-1p_growth_28hr	0.2 (within 100800 s)			Likelihood of T cell division for PD-1+ T cell
PD-1n_migration	10 μm/min	[10]		Migration speed for PD-1- T cell
PD-1p_migration	5 μm/min			Migration speed for PD-1+ T cell
LymphNode_delay_growth	32400 sec	[11]		T cells in lymph node divide 5-6 times in 24 hours
migration_MHCIp_tumor_dwell_velocity	0 μm/min	[12]		T cells slow down when engaging with MHCI+ on tumor cells
PD-1n_migration_MHCIp_tumor_dwell_time	25 min			Duration for which PD-1- T cells slow down during engagement with MHCI+ on tumor cells
PD-1p_migration_MHCIp_tumor_dwell_time	10 min			Duration for which PD-1+ T cells slow down during engagement with MHCI+ on tumor cells
PD-1n_migration_refractory_time	35 min			Duration for which PD-1- T cells do not interact with tumor

				cells following an engagement with MHCI+ tumor cell (10 minutes)
PD-1p_migration_refractory_time	20 min			Duration for which PD-1- T cells do not interact with tumor cells following an engagement with MHCI+ tumor cell (10 minutes)
cytotoxic_packet_production	40 /min	[13,14]		Number of cytotoxic packets produces per T cell
PD-1n_cytotoxic_packets_max	10000			Maximum number of cytotoxic packets a PD-1- T cell can produce
cytotoxic_transfer_rate	400 /min[1]			Maximum transfer rate of cytotoxic packets from T cell to Tumor
PD-1p_cytotoxic_packets_max	1000 (counts)	[7]		Maximum number of cytotoxic packets a PD-1+ T cell can produce
MHCIn_reduction_production	400	[15,16]		4-fold reduction in production in T cells in contact with MHCI- tumor*
diameter	15 μm	Multiplexed Imaging Data	tumor.py	Average diameter of a tumor cell
initial_PDL1n, cell_state	Vary	Multiplexed Imaging Data		Based on percentage of cells that are PDL1- when transferred in
Location [x, y]	Vary	Multiplexed Imaging Data		Spatial position of tumor cells in simulation
MHCI	Vary	Multiplexed Imaging Data		Total protein expressed of MHCI on tumor cells
PDL1	Vary	Multiplexed Imaging Data		Total protein expressed of PDL1 on tumor cells
IFNg	Vary	Multiplexed Imaging Data		Total IFNg received in tumor cells

[1] Note that cytotoxic packets are all multiplied by 100 to deal with timestep of 60 seconds since actual value is 0.4/minute

death_apoptosis	0.5 (within 432000 s)	[17]		Likelihood of tumor cell death based on apoptosis
cytotoxic_packet_threshold	128 (counts)	[13,14]		Number of cytotoxic packets required for a tumor cell to receive to induce death
PDL1n_growth	0.6 (within 86400 s)	[18]		Likelihood of tumor cell division for PDL1- tumor cell
Max_IFNg_internalization	31 molecules/min	[19]		Maximum number of IFNg molecules a tumor cell can internalize
IFNg_threshold	15000 molecules	[12,20]		Number of IFNg molecules needed to induce phenotype switching in tumor cells
IFNg_MW	17000 g/mol			IFNg molecular weight
reduction_IFNg_internalization	2	[21,22]		Fold reduction in uptake by PDL1+ tumor cells
tumor_debris_amount	1.4e15 (counts for HGB1)	[23]		Number of apoptotic molecules released by dying tumor cells
Diameter	10 μm	[24]	dendritic_cell.py	Average diameter of a dendritic cell
Location [x, y]	Vary	Multiplexed Imaging Data		Spatial position of dendritic cells in simulation
velocity	3 μm/min	[25]		Cell migration speed for dendritic cells
death_apoptosis	0.5 (within 4 days)	[26]		Likelihood of dendritic cell death based on apoptosis
divide_prob	0.5 (within 5 days)	In vitro data		Likelihood of tumor cell division for dendritic cell
divide_time	5 days			Period over the likelihood of dendritic cell division
internal_tumor_debris_threshold	415,000 (counts)	[27]		Number of apoptotic molecules required for dendritic cell activation
tumor_debris_uptake	300 molecules/cell/hr			Maximum number of apoptotic molecules a dendritic cell can internalize

tumor_debris_MW	29,000 g/mol (assuming for HGMB1)	[23]		Apoptotic molecule molecular weight (used for diffusion)
n_tcells_in_lymph_node	3 (assume size for 1200 tumor)	Multiplexed Imaging Data	lymph_node.py	Number of antigen-specific T cells found within the lymph node
tcell_find_dendritic_time	0.95 (within 14400 s)	[28,29]		Percentage of T cells that will find dendritic cells within expected interval period
expected_dendritic_transit_time	28800 s			Average amount of time it takes for a dendritic cell to travel from tumor to lymph node
expected_interaction_duration	28800 s			Average amount of time T cells interact and get activated by dendritic cells in lymph node
expected_delay_before_migration	43200 s			Average amount of time that T cells reside in lymph node post dendritic cell engagement
expected_tcell_transit_time	3600 s	[30]		Average amount of time that it takes for activated T cells to migrate from lymph node to the tumor
DIFFUSION_RATES: IFNg	1.25e-3 cm^2/day	[31]	fields.py	Diffusion rate for IFNg
DIFFUSION_RATES: tumor_debris	0.0864 cm^2/day	[33]		Diffusion rate for apoptotic tumor debris molecules
decay: IFNg	4.5 hr	[32]		Decay rate for IFNg in the tumor environment

Supplemental Table 2: Excel file with CyTOF and CODEX antibody panels for characterizing cellular metabolism and spatial cellular distributions.

REFERENCES

1. Zhao, M., Kiernan, C.H., Stairiker, C.J., Hope, J.L., Leon, L.G., van Meurs, M., Brouwers-Haspels, I., Boers, R., Boers, J., and Gribnau, J. (2020). Rapid in vitro generation of bona fide exhausted CD8+ T cells is accompanied by Tcf7 promotor methylation. PLoS Pathog. *16*, e1008555.

2. Salerno, F., Paolini, N.A., Stark, R., von Lindern, M., and Wolkers, M.C. (2017). Distinct PKC-mediated posttranscriptional events set cytokine production kinetics in CD8+ T cells. Proc. Natl. Acad. Sci. *114*, 9677–9682.
3. Gallegos, A.M., Xiong, H., Leiner, I.M., Sušac, B., Glickman, M.S., Pamer, E.G., and van Heijst, J.W.J. (2016). Control of T cell antigen reactivity via programmed TCR downregulation. Nat. Immunol. *17*, 379–386.
4. Petrovas, C., Price, D.A., Mattapallil, J., Ambrozak, D.R., Geldmacher, C., Cecchinato, V., Vaccari, M., Tryniszewska, E., Gostick, E., and Roederer, M. (2007). SIV-specific CD8+ T cells express high levels of PD-1 and cytokines but have impaired proliferative capacity in acute and chronic SIVmac251 infection. Blood, J. Am. Soc. Hematol. *110*, 928–936.
5. Tang, X., Li, Q., Zhu, Y., Zheng, D., Dai, J., Ni, W., Wei, J., Xue, Y., Chen, K., and Hou, W. (2015). The advantages of PD-1 activating chimeric receptor (PD-1-ACR) engineered lymphocytes for PDL1+ cancer therapy. Am. J. Transl. Res. *7*, 460.
6. Dong, H., Strome, S.E., Salomao, D.R., Tamura, H., Hirano, F., Flies, D.B., Roche, P.C., Lu, J., Zhu, G., and Tamada, K. (2002). Tumor-associated B7-H1 promotes T-cell apoptosis: a potential mechanism of immune evasion. Nat. Med. *8*, 793–800.
7. Zelinskyy, G., Robertson, S.J., Schimmer, S., Messer, R.J., Hasenkrug, K.J., and Dittmer, U. (2005). CD8+ T-cell dysfunction due to cytolytic granule deficiency in persistent Friend retrovirus infection. J. Virol. *79*, 10619–10626.
8. Bouchnita, A., Bocharov, G., Meyerhans, A., and Volpert, V. (2017). Hybrid approach to model the spatial regulation of T cell responses. BMC Immunol. *18*, 1–12.
9. Vodnala, S.K., Eil, R., Kishton, R.J., Sukumar, M., Yamamoto, T.N., Ha, N.-H., Lee, P.-H., Shin, M., Patel, S.J., and Yu, Z. (2019). T cell stemness and dysfunction in tumors are triggered by a common mechanism. Science (80-.). *363*, eaau0135.
10. Boissonnas, A., Fetler, L., Zeelenberg, I.S., Hugues, S., and Amigorena, S. (2007). In vivo imaging of cytotoxic T cell infiltration and elimination of a solid tumor. J. Exp. Med. *204*, 345–356.
11. Mempel, T.R., Henrickson, S.E., and Von Andrian, U.H. (2004). T-cell priming by dendritic cells in lymph nodes occurs in three distinct phases. Nature *427*, 154–159.
12. Thibaut, R., Bost, P., Milo, I., Cazaux, M., Lemaître, F., Garcia, Z., Amit, I., Breart, B., Cornuot, C., and Schwikowski, B. (2020). Bystander IFN-γ activity promotes widespread and sustained cytokine signaling altering the tumor microenvironment. Nat. Cancer *1*, 302–314.
13. Betts, M.R., and Koup, R.A. (2004). Detection of T-cell degranulation: CD107a and b. Methods Cell Biol. *75*, 497–512.
14. Zhang, M., Park, S.-M., Wang, Y., Shah, R., Liu, N., Murmann, A.E., Wang, C.-R., Peter, M.E., and Ashton-Rickardt, P.G. (2006). Serine protease inhibitor 6 protects cytotoxic T cells from self-inflicted injury by ensuring the integrity of cytotoxic granules. Immunity *24*, 451–461.
15. Böhm, W., Thoma, S., Leithäuser, F., Möller, P., Schirmbeck, R., and Reimann, J. (1998). T cell-mediated, IFN-γ-facilitated rejection of murine B16 melanomas. J. Immunol. *161*, 897–908.
16. Merritt, R.E., Yamada, R.E., Crystal, R.G., and Korst, R.J. (2004). Augmenting major histocompatibility complex class I expression by murine tumors in vivo enhances antitumor immunity induced by an active immunotherapy strategy. J. Thorac. Cardiovasc. Surg. *127*, 355–364.
17. Gong, C., Milberg, O., Wang, B., Vicini, P., Narwal, R., Roskos, L., and Popel, A.S. (2017). A computational multiscale agent-based model for simulating spatio-temporal tumour immune response to PD-1 and PDL1 inhibition. J. R. Soc. Interface *14*, 20170320.

18. Eden, E., Geva-Zatorsky, N., Issaeva, I., Cohen, A., Dekel, E., Danon, T., Cohen, L., Mayo, A., and Alon, U. (2011). Proteome half-life dynamics in living human cells. Science (80-.). *331*, 764–768.
19. Celada, A., and Schreiber, R.D. (1987). Internalization and degradation of receptor-bound interferon-gamma by murine macrophages. Demonstration of receptor recycling. J. Immunol. *139*, 147–153.
20. Hoekstra, M.E., Bornes, L., Dijkgraaf, F.E., Philips, D., Pardieck, I.N., Toebes, M., Thommen, D.S., van Rheenen, J., and Schumacher, T.N.M. (2020). Long-distance modulation of bystander tumor cells by CD8+ T-cell-secreted IFN-γ. Nat. Cancer *1*, 291–301.
21. El Darzi, E., Bazzi, S., Daoud, S., Echtay, K.S., and Bahr, G.M. (2017). Differential regulation of surface receptor expression, proliferation, and apoptosis in HaCaT cells stimulated with interferon-γ, interleukin-4, tumor necrosis factor-α, or muramyl dipeptide. Int. J. Immunopathol. Pharmacol. *30*, 130–145.
22. Ersvaer, E., Skavland, J., Ulvestad, E., Gjertsen, B.T., and Bruserud, Ø. (2007). Effects of interferon gamma on native human acute myelogenous leukaemia cells. Cancer Immunol. Immunother. *56*, 13–24.
23. Apetoh, L., Ghiringhelli, F., Tesniere, A., Obeid, M., Ortiz, C., Criollo, A., Mignot, G., Maiuri, M.C., Ullrich, E., and Saulnier, P. (2007). Toll-like receptor 4–dependent contribution of the immune system to anticancer chemotherapy and radiotherapy. Nat. Med. *13*, 1050–1059.
24. Morefield, G.L., Sokolovska, A., Jiang, D., HogenEsch, H., Robinson, J.P., and Hem, S.L. (2005). Role of aluminum-containing adjuvants in antigen internalization by dendritic cells in vitro. Vaccine *23*, 1588–1595.
25. Lämmermann, T., Bader, B.L., Monkley, S.J., Worbs, T., Wedlich-Söldner, R., Hirsch, K., Keller, M., Förster, R., Critchley, D.R., and Fässler, R. (2008). Rapid leukocyte migration by integrin-independent flowing and squeezing. Nature *453*, 51–55.
26. Naik, S.H. (2008). Demystifying the development of dendritic cell subtypes, a little. Immunol. Cell Biol. *86*, 439–452.
27. Yang, D., Chen, Q., Yang, H., Tracey, K.J., Bustin, M., and Oppenheim, J.J. (2007). High mobility group box-1 protein induces the migration and activation of human dendritic cells and acts as an alarmin. J. Leucoc. Biol. *81*, 59–66.
28. Bousso, P. (2008). T-cell activation by dendritic cells in the lymph node: lessons from the movies. Nat. Rev. Immunol. *8*, 675–684.
29. Itano, A.A., and Jenkins, M.K. (2003). Antigen presentation to naive CD4 T cells in the lymph node. Nat. Immunol. *4*, 733–739.
30. Hunter, M.C., Teijeira, A., and Halin, C. (2016). T cell trafficking through lymphatic vessels. Front. Immunol. *7*, 613.
31. Liao, K.-L., Bai, X.-F., and Friedman, A. (2014). Mathematical modeling of interleukin-27 induction of anti-tumor T cells response. PLoS One *9*, e91844.
32. Kurzrock, R., Rosenblum, M.G., Sherwin, S.A., Rios, A., Talpaz, M., Quesada, J.R., and Gutterman, J.U. (1985). Pharmacokinetics, single-dose tolerance, and biological activity of recombinant γ-interferon in cancer patients. Cancer Res. *45*, 2866–2872.
33. Krouglova, T., Vercammen, J., and Engelborghs, Y. (2004). Correct diffusion coefficients of proteins in fluorescence correlation spectroscopy. Application to tubulin oligomers induced by Mg2+ and Paclitaxel. Biophys. J. *87*, 2635–2646.

Supplemental Text

Here we provide more extensive background about the model development, parameters, processes, deployment, and the Vivarium software infrastructure. This document is organized to reflect how the python files are organized within the repository in order to improve readability and associated documentation. The code is freely available at this GitHub repository: https://github.com/vivarium-collective/tumor-tcell. Throughout this supplemental text we will describe the different files, objects, and methods that encode the different model processes, composites and simulation experiments. All of the diagrams in this supplement were programmatically generated from the source experiment, and the code can be found in a "diagrams.ipynb" notebook in the tumor-tcell GitHub repository: https://github.com/vivarium-collective/tumor-tcell/blob/master/jupyter_notebooks/diagrams.ipynb

Vivarium Overview

Vivarium is a versatile software tool that offers an interface for simulation "process" modules (**Fig. S1A**), which can be connected to stores that hold the system states, which could be of any datatype (float, array, or more complex structure). These can be combined into composite hybrid simulations, parallelized across computing nodes, and co-simulated across multiple spatial and temporal scales. Its hierarchical structure (**Fig. S1B**) supports nesting and multi-scale representations, including agent-based models. These are co-simulation by Vivarium's discrete-event simulation engine (**Fig. S1C**). Distinguished from monolithic simulators, Vivarium's modular approach promotes interoperability, reusability, and composability. Its plug-in system reduces the barrier to contribution, making it simpler to integrate novel processes. This supplement introduces the specific Vivarium processes and composites used for the Tumor/ T cell simulation. For details on the Vivarium methodology, see the Vivarium paper[1] and accompanying documentation.

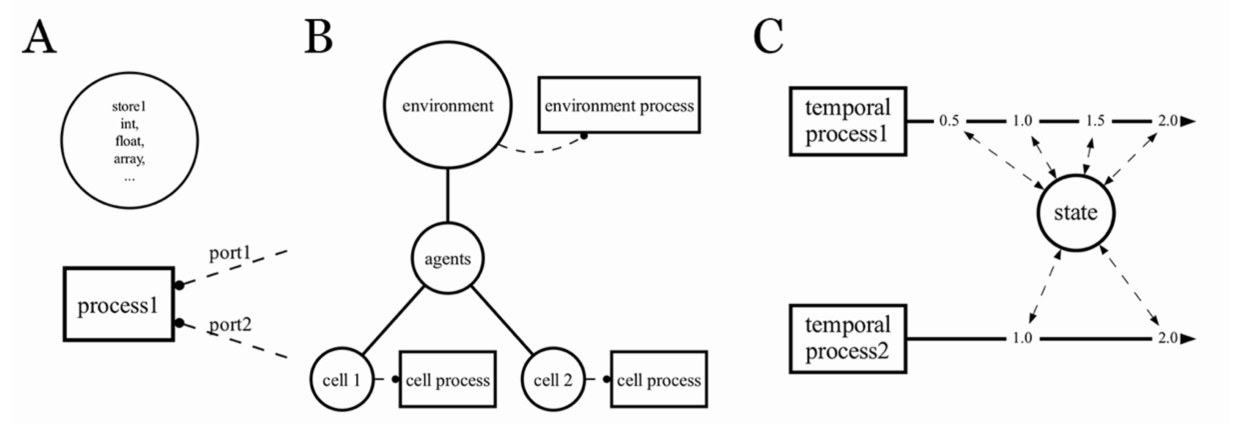

Figure S1. Vivarium basic concepts. A) Vivarium's units are processes and stores. Processes wrap around simulation modules, exposing an interface made of ports. Stores hold the system variables and allow for easy linking with other processes – these can include any type of data, whether it is a stream of numbers of more complex data. These are structured in a hierarchy, which allows for multiscale representation. B) A Composite is a hybrid simulator, defined by a wiring specification and constructed by the Vivarium engine. This one shows a simple agent-based model schema with cells that have their own processes, in an environment that has one process. C) The engine the orchestrates the processes, using a discrete-event simulation algorithm to trigger the processes according to their preferred synchronization time steps.

Tumor-Tcell ABM

The rest of the supplement goes into the specific implementations used for the paper. First, we look at the full simulation experiment as a composite, then we look at sub-composites and their individual processes, including equations and parameters. This is done first for environmental processes, and then cellular processes.

- The main simulations can be found at: **`tumor_tcell/experiments/main.py`**
- All processes can be found at: **`tumor_tcell/processes/`**
- All composites can be found at: **`tumor_tcell/composites/`**

Experiments

A Vivarium diagram of the final simulation configuration is shown in **Fig S2**, collapsing the internal state of the lymph node and in_transit stores for visual simplification, the tumor_environment includes only three cells which are also simplified by removing many internal states. But as you can see the graph is already fairly complex. Fig S3 shows an even further simplified simulation configuration, with only one cell and many collapsed nodes. This is constructed programmatically from an experiment generated within the "main" python file, which can be found at the path: **`tumor_tcell.experiments.main.py`**

The main file includes all the configurations and functions used to generate and run the paper's simulation experiments. The main method is **`` `tumor_tcell_abm()` ``**, which takes various system inputs, including the size of the tumor microenvironment, time step, initial agent counts (tumors, T cells, dendritic cells) and phenotype with ratios, agent interactions and environmental factors. Agents are placed within the simulation environment, and their states are initialized. Simulation results, including agent states and environmental factors, is returned by the method. The file includes functions for generating plots and visualizing simulation results, including multi-generation timeseries plots for T cells and tumors, snapshots of the simulation at different time points, and video generation. Different workflows define sets of experiments and plots that can be triggered from the command line or as imports into a different python file.

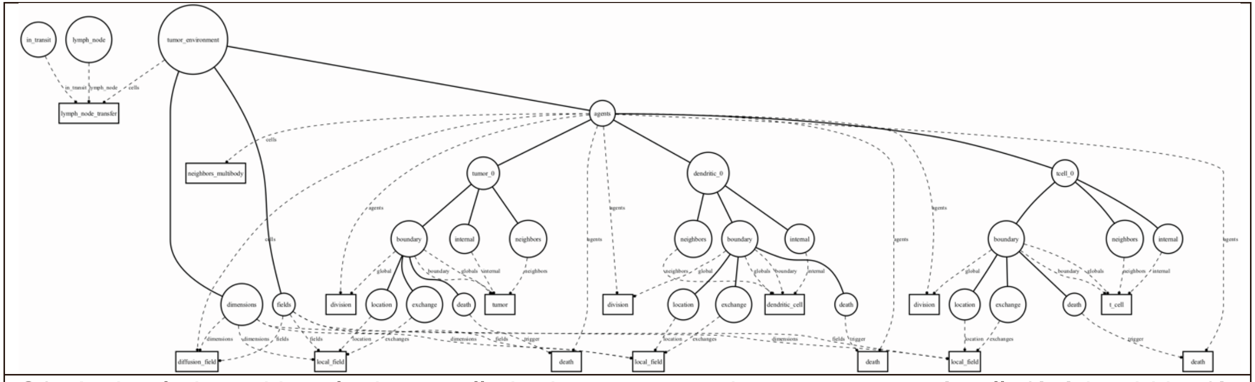

S2. A simulation with only three cells in the tumor_environment store –`tcell_0`, `dendritic_0`, and `tumor_0'. No cells are shown in the lymph_node or in_transit as it would have made this figure even harder to read – imagine the complexity of a full simulation with hundreds to thousands of cells across all three locations. The figure S3 below simplifies the structure further for legibility and adds more descriptions.

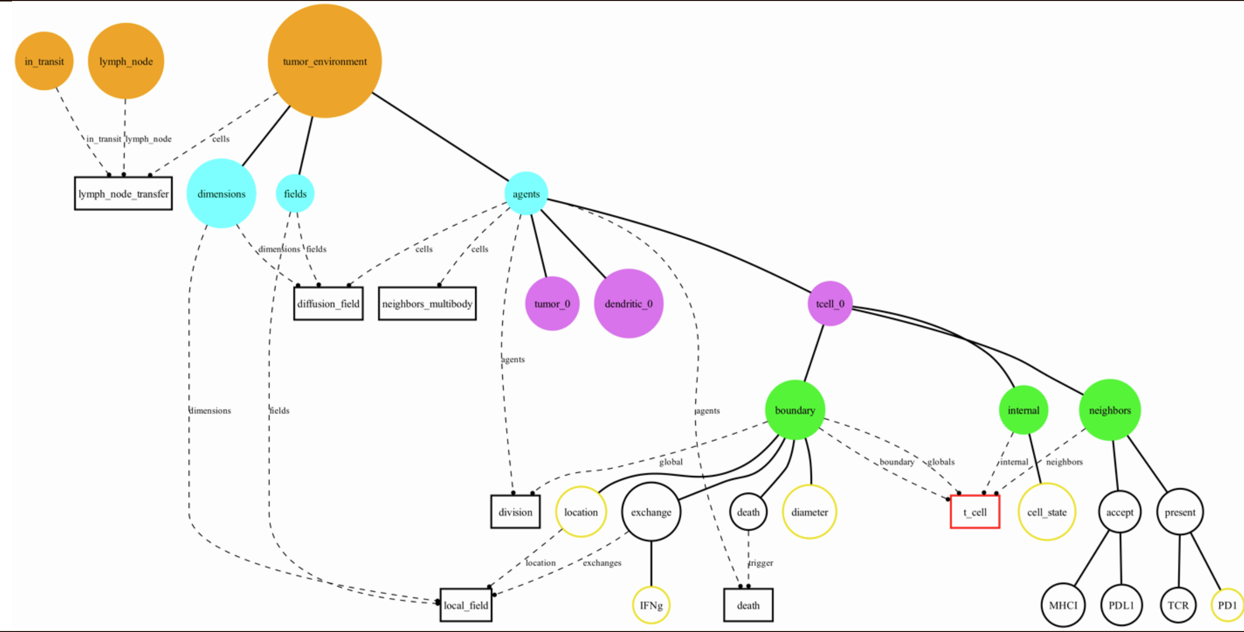

Fig S3. A piece of the full agent-based model used in this paper, collapsing some details. Top-level stores, shown in orange, include the tumor_microenvironment, in_transit and lymph_node, these are updated by the lymph_node_transfer process, which is described below. Within the tumor_environment there are store agents, fields, and dimensions, which are updated by diffusion_field and neighbors_multibody in spatial environmental simulation. Within the agents there are three cells, shown in purple, tumor_0 and dendritic_0 are fully collapsed, while tcell_0 shows some detail but also this is incomplete for simplicity. Some of the T cell top-level internal state are shown in green – neighbors, internal, and boundary. There are a number of internal processes, but the main one – t_cell – is shown outlined in red. States initialized from data are shown outlined in yellow. For more detailed information of what data was used as parameters or input see the parameter tables within each section.

Probability functions

Many actions are underpinned by time-bound probability functions. For instance, rates of cell death and division—barring deaths caused by T cells' cytotoxic packets—are determined by expected values sourced from lab findings and literature, contingent upon environmental cues. The two functions are common across processes for this:

1. P will represent the method **`get_probability_timestep(probability_parameter, timescale, timestep)`** found in the code. It computes the transition probability for a specific timestep based on an initial probability and a defined timescale. The parameters are:
   - **`probability_parameter`**: The initial probability of an event occurring within the defined timescale.
   - **`timescale`**: The observation period for the transition probability.
   - **`timestep`**: The specific time interval for calculating the transition probability.

Using these, the function first determines the rate of the exponential decay, and then the transition probability at the desired timestep δ using the formula:

$$P(\delta) = 1 - e^{-\ln(1-p)*\frac{\delta}{T}}$$

where e is the base of natural logarithms, p is the probability parameter, T is the timescale, and δ is the timestep.

2. U will represent the method **`probability_of_occurrence_within_interval( interval_duration, expected_time)`** found in the code, which computes the probability that an event will occur at least once within a specified time interval, assuming the event adheres to a Poisson process. The parameters are:
   - **`interval_duration`**: The time duration over which you wish to determine the likelihood of an event happening at least once.
   - **`expected_time`**: The mean time between successive events, representing the average duration for the event to transpire.

From these inputs, the function derives the rate parameter λ by the relation:

$$\lambda = \frac{\text{interval}}{\text{expected\_time}}$$

indicating the mean number of events expected within the interval. It then evaluates the probability of no events within the interval as

$$\mathrm{P_0} = \mathrm{e}^{-\lambda}$$

where e is the base of natural logarithms. The probability of at least one event within the specified interval δ is given by:

$$U(\delta) = 1 - P_0$$

Environmental Processes

First, we look at the composites and processes the simulate the environment.

Lymph node process
(tumor_tcell.processes.lymph_node.py)

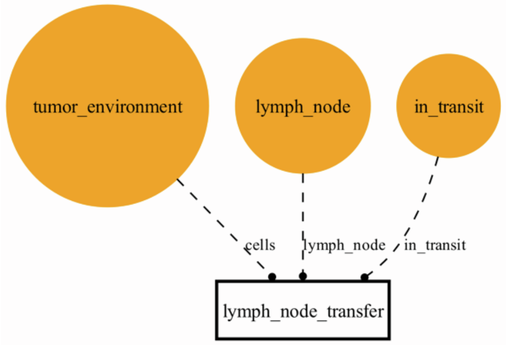

This process simulates the movement between 3 main compartments – a tumor environment, a lymph node, and the transit route (vasculature) between the tumor and lymph node. The process also simulates interaction of dendritic and T cells within the lymph node.

This model assumes there are some number of T cells in the lymph node that are antigen-specific for the tumor. If there are any dendritic cells present within the lymph node, then the probability

of the T cell initializing an interaction with dendritic cells is determined. This probability is based on the expected time for T cells to find dendritic cells. A random number will determine if the event happens, triggering T cell transitions to the 'interacting' state, simulating the start of an interaction with dendritic cells. Similarly, if the T cell is in the 'interacting' state, it represents an ongoing interaction with dendritic cells that is terminated once at a given probability and transitions to a 'delay' state, which then also follows this same procedure to then move to 'in_transit.' Both T cells and dendritic cells spend time 'in_transit', which captures the migration time of dendritic cells from the tumor microenvironment within lymphatics and T cells to the tumor through the vasculature.

Lymph Node Behavior: For T cells within the lymph node, the **`get_probability_timestep`** method, P, is used with the parameters below to determine the probability of completion if they are in an 'interacting' state, whether they begin migration to the tumor if they are in a 'delay' state, and the T cell's probability of interacting with available dendritic cells if they are not 'interacting' or in 'delay'.

Tumor Environment Behavior: If dendritic cells turn into the 'active' state, they move to in transit.

In Transit Behavior: Cells that are "in transit" are considered to be going through the vasculature, either from the lymph node to the tumor or the other way around. Dendritic cells' probability of arriving at the lymph node and T cells' probability of arriving at the tumor all use the **`probability_of_occurrence_within_interval`** method U with the expected time durations listed in the parameter table below.

Here are parameters loaded within **`lymph_node.py`** and the source of where they are coming from and whether they were varied within the experiments:

Parameter name	Value	Source	Location
n_tcells_in_lymph_node	3 (assume size for 1200 tumor)	Multiplexed Imaging Data	lymph_node.py
tcell_find_dendritic_time	0.95 (within 14400 s)	[2,3]	
expected_dendritic_transit_time	28800 s		
expected_interaction_duration	28800 s		
expected_delay_before_migration	43200 s		
expected_tcell_transit_time	3600 s	[4]	

Tumor microenvironment composite
(`tumor_tcell.composites.tumor_microenvironment.py`)

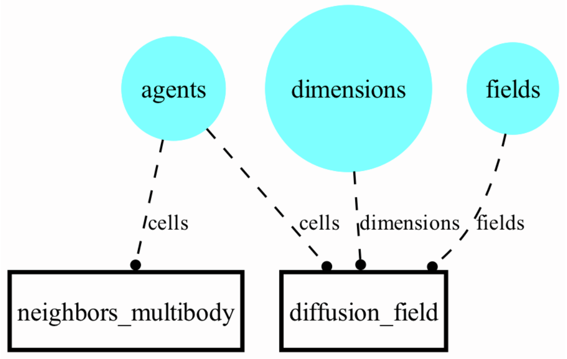

This is composite simulates a 2D environment with agents that can move around in space, exchange molecules with their neighbors, and exchange molecules with a molecular field. This includes a **`Neighbors`** process, which tracks the locations of individual agents and handles their exchanges, and a diffusion **`Fields`** process, which operates on the molecular fields. In the full simulation, it lives only within the "tumor environment" store – the lymph node uses a non-spatial environment.

Neighbors Process
(tumor_tcell.processes.neighbors.py)

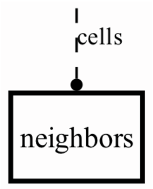

This process represents a multibody physics process with neighbor tracking, simulating collisions between cell bodies and managing the exchange of molecules between these cells.

The physics component of the process has each agent (cell) characterized by its physical properties, including location, diameter, mass, and velocity. **`diameter`, `mass` and `velocity`** can be updated by individual cells internal processes, which **`location`** is handled by the neighbors process alone as determined by the physics simulation. For the physics, this process uses pymunk (http://www.pymunk.org/), which models individual cell agents as circular rigid bodies that can move, grow, and collide. This includes random jitter to model Brownian motion, and friction to model cell-cell adhesion. For more information on the meaning of the elasticity and friction parameters, see the pymunk documentation. The use of pymunk was also described in the Vivarium paper[1].

In addition to physics, the Neighbors process handles the exchange of molecules between cells, through the ports **`transfer`, `receive`, `accept`**, and **`present`,** each of which represents a different aspect of intercellular communication. For soluble signaling molecules, **`transfer`** and **`receive`** represent giving and receiving molecules from neighboring cells, respectively. For membrane-bound signaling molecules, **`accept`** represents the biological response to the binding action. **`present`** signifies the ligand, which triggers a biological response for cells with matching **`accept`**. We define neighboring cells with the function called **`get_neighbors`,** which takes cell coordinates and cell radius and finds neighboring cell ID with its radius.

Fields process
(tumor_tcell.processes.fields.py)

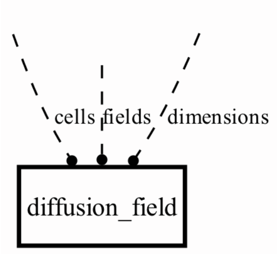

This process models the diffusion and decay of molecular concentrations in a 2D field. The 'Fields' class is initialized with various parameters that define the characteristics of the field, such as its size, resolution, and the properties of the molecules being simulated. It has previously been described[1].

The key parameters used in the process include:
- **`bounds`**: specifies the size of the environment in micrometers along the x and y dimensions.
- **`n_bins`**: defines the resolution of the environment by the number of bins in both x and y directions within the environment bounds.
- **`depth`**: represents the depth of the field.
- **`molecules`**: A list of the types of molecules being simulated.
- **`default_diffusion_dt`**: The time step used for diffusion calculations.
- **`default_diffusion_rate`**: The default diffusion rate for molecules.
- **`diffusion`**: Specific diffusion rates for individual molecules.
- **`decay`**: Specific decay rates for molecules, determines how molecules degrade over time.

Diffusion and decay: For each molecular type, the concentration undergoes an exponential decay based on a decay rate k. The decayed concentration F' after a time step Δt is given by:

$$F' = F_0 \times e^{-k\Delta t}$$

Diffusion is achieved by applying the 2D Laplacian kernel over a field post decay, F', representing molecular concentration. This convolution captures the spatial variation of molecules within the field. The field is then updated to its final state, F'', by multiplying the outcome with a diffusion constant D and a time step Δt.

$$F'' = F' + D\Delta t \cdot F' * \begin{pmatrix} 0 & 1 & 0 \\ 1 & -4 & 1 \\ 0 & 1 & 0 \end{pmatrix}$$

Here are parameters loaded within **`fields.py`** and the source of where they are coming from and whether they were varied within the experiments:

Parameter name	Value	Source	Location
DIFFUSION_RATES: IFNg	1.25e-3 cm^2/day	[5]	fields.py
DIFFUSION_RATES: tumor_debris	0.0864 cm^2/day	[6]	
decay: IFNg	4.5 hr	[7]	

Local Field Process
(tumor_tcell.processes.local_field.py)

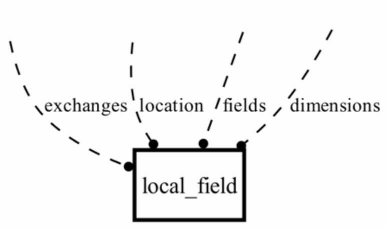

`LocalField` belongs to individual cells but is placed here along with the environmental processes because it serves as an adapter between two-dimensional array for the environment to each cell's local environment state representation. It allows individual cell agents to uptake and release molecules at their specific locations within the field.

Cell Processes

Cell composites (**Fig S4)** are integrated models with multiple initialized processes, which can plug into the environment's "agents" state as cellular agents. Each cell composite has a core cell process combined with additional helper processes such as death, division, and local field. The division process waits for the division flag and then carries out division, the death process waits for a death flag and then removes the agent, and a local field, which was described above, interfaces the external environment to support uptake and secretion. This section focuses on the core cell processes for T cells, dendritic cells, and tumor cells.

Each composite cell agent can be found at these locations:
- **`tumor_tcell.composites.t_cell_agent.py`**
- **`tumor_tcell.composites.dendritic_agent.py`**
- **`tumor_tcell.composites.tumor_agent.py`**

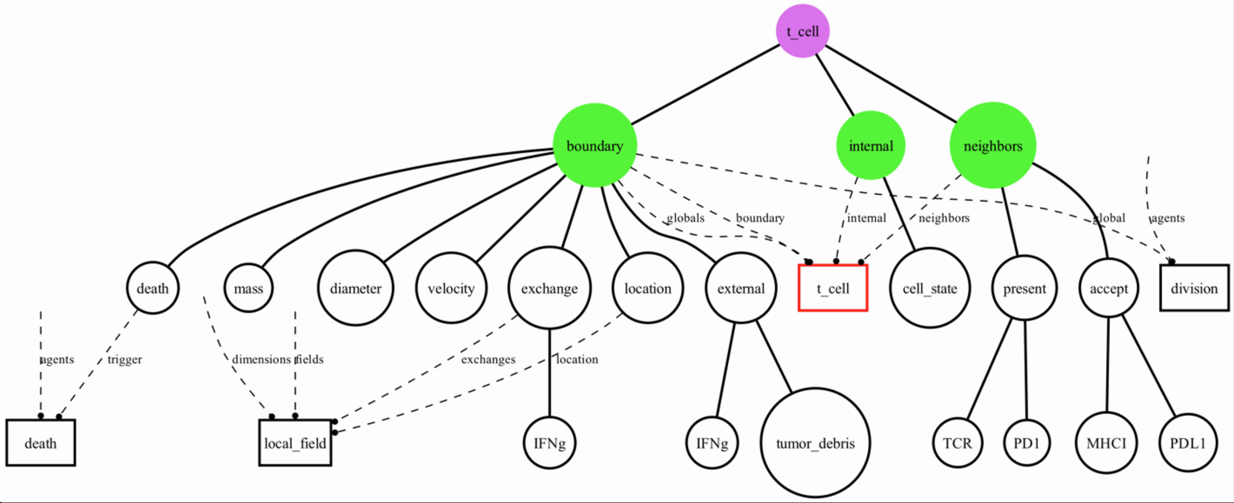

Fig S4. A T Cell Agent Composite. The T cell core process is shown with a red border. Additional helper processes are local_fields, death, and division. These connect the t_cell agent to the higher-scale agents and fields stores, here shown as disconnected ports pointing up.

T cell process
(`tumor_tcell.processes.t_cell.py`)

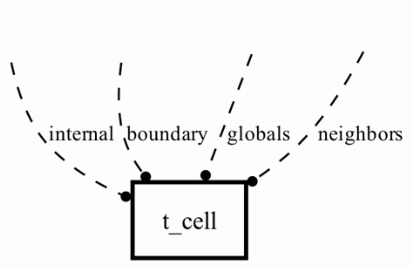

The model illustrates the lifecycle and behavior of T cells, which predominantly exist in two states:

1. PD1n (PD1-negative): In this state, T cells are active and have a high capacity to interact with tumor cells and other antigens.
2. PD1p (PD1-positive): Here, T cells are less active and their functionality might be suppressed due to prolonged activation or interactions with PDL1.

T cells transition between these states based on conditions like the duration of their activation and the number of divisions they've undergone. They can undergo apoptosis, especially when they've been activated for too long or have been suppressed by interactions with PDL1 on tumor cells. T cells can kill tumor cells if they release enough cytotoxic packets upon them.

Mechanisms:
1. Death by Apoptosis for PD1, and for PDL1 are both determined by the **`get_probability_step`** method, P, using the parameters "death_PD1n_14hr" and "death_PD1p_next_to_PDL1_14hr". These parameters are reported probabilities, the method gives us the probability of occurrence within the given timestep δ. A random number in the range (0, 1) determines death.
2. IFNγ Production: T cells produce interferon-gamma (IFNγ) when activated. The rate of IFNγ production is constant based on which state it is in, based on whether it is interact with MHCI molecules on tumor cells.

Here are parameters loaded within **`t_cell.py`** and the source of where they are coming from and whether they were varied within the experiments:

Parameter name	Value	Source	Location
diameter	7.5 μm	Multiplexed Imaging Data	t_cell.py
initial_PD1n, cell_state	Vary	Multiplexed Imaging Data, CyTOF data	
Location [x, y]	Vary	Multiplexed Imaging Data	
PD1	Vary	Multiplexed Imaging Data	
IFNg	Vary	CyTOF Data	
PDL1_critical_number, ligand_threshold	1e4 (count/cell)	[8]	
TCR_downregulated	0		
TCR_upregulated	50000 (count/cell)		
refractory_count_threshold	3 (assuming stimulated 2 during in vitro activation)		
PD1n_divide_threshold	5		
activation_time	21600 s	[9,10]	
activation_refractory_time	43200 s		
death_PD1p_14hr	0.35 (within 50400 s)	[11]	

death_PD1n_14hr	0.1 (within 50400 s)		
death_PD1p_next_to_PDL1p_14hr	0.475 (within 50400 s)	[12,13]	
PD1n_IFNg_production	1.62e4 molecules/cell/s	[14,15]	
PD1p_IFNg_production	1.62e3 molecules/cell/s		
PD1n_growth_28hr	0.9 (within 100800 s)	[11,16]	
PD1p_growth_28hr	0.2 (within 100800 s)		
PD1n_migration	10 μm/min	[17]	
PD1p_migration	5 μm/min		
LymphNode_delay_growth	32400 sec	[18]	
migration_MHCIp_tumor_dwell_velocity	0 μm/min	[19]	
PD1n_migration_MHCIp_tumor_dwell_time	25 min		
PD1p_migration_MHCIp_tumor_dwell_time	10 min		
PD1n_migration_refractory_time	35 min		
PD1p_migration_refractory_time	20 min		
cytotoxic_packet_production	40 /min	[20,21]	
PD1n_cytotoxic_packets_max	10000		
cytotoxic_transfer_rate	400 /min		
PD1p_cytotoxic_packets_max	1000 (counts)	[14]	
MHCIn_reduction_production	400	[22,23]	

Tumor cell process
(`tumor_tcell.processes.tumor.py`)

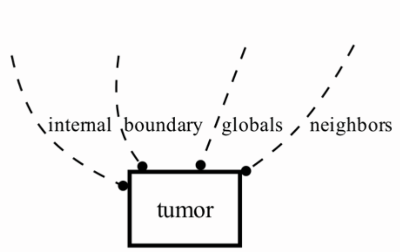

The model depicts the lifecycle and behavior of tumor cells, which exist primarily in two states:

1. **Proliferative state**: Here, the tumor cells have a low presence of immune molecules (MHCI and PDL1).
2. **Quiescent state**: In this state, the tumor cells exhibit elevated levels of immune molecules (MHCI and PDL1).

Tumor cells transition between these states based on their exposure to IFNg from T cells. Furthermore, they can be removed either through natural processes like apoptosis or when attacked by T cells, given the release of cytotoxic packets.

Mechanisms:

1. Death by Apoptosis: which represents the chance of a tumor cell undergoing natural death uses `get_probability_step` method, P, using the parameters "death_apoptosis" for timescale 5 days. If this is triggered, the tumor cell dies and releases debris.
2. Death by T-cell: Tumor cells can be killed by immune cells called T cells. If the number of cytotoxic packets received from T cells surpasses a threshold, the tumor cell is killed and sheds debris.
3. IFNg Uptake: The model calculates available IFNg by determining how far it can diffuse in a timestep and then computing the volume it can reach around the tumor. This volume helps determine how much IFNg the tumor can access.

$$\text{Diffusion\_Radius}=\text{Diffusion\_Rate}\times\text{Timestep}$$
$$\text{Available\_Volume}=\frac{4}{3}\pi\times(\text{Tumor\_Radius}+\text{Diffusion\_Radius})^3$$
$$\text{Available\_IFNg}=\text{External\_IFNg}\times\text{Available\_Volume}$$

Here are parameters loaded within `tumor.py` and the source of where they are coming from and whether they were varied within the experiments:

Parameter name	Value	Source	Location
diameter	15 μm	Multiplexed Imaging Data	tumor.py
initial_PDL1n, cell_state	Vary	Multiplexed Imaging Data	
Location [x, y]	Vary	Multiplexed Imaging Data	
MHCI	Vary	Multiplexed Imaging Data	
PDL1	Vary	Multiplexed Imaging Data	
IFNg	Vary	Multiplexed Imaging Data	
death_apoptosis	0.5 (in 5 days)	[24]	
cytotoxic_packet_threshold	128 (counts)	[20,21]	
PDL1n_growth	0.6 (within 86400 s)	[25]	
Max_IFNg_internalization	31 molecules/min	[26]	
IFNg_threshold	15000 molecules	[19,27]	
IFNg_MW	17000 g/mol		
reduction_IFNg_internalization	2	[28,29]	
tumor_debris_amount	1.4e15 (counts for HGB1)	[30]	

Dendritic cell process
(`tumor_tcell.processes.dendritic_cell.py`)

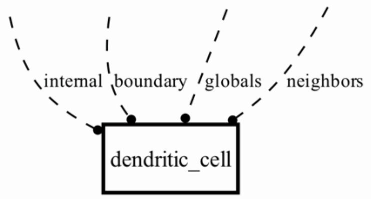

Dendritic cells (DCs) are pivotal in the immune system, primarily facilitating antigen presentation and steering the immune response. The model captures diverse behaviors of DCs, including apoptosis, division, state transitions, and their interactions with tumor debris, T cells, and migration patterns towards lymph nodes. Key aspects include:

- **Inactive-to-Active Transition:** DCs shift from "inactive" to "active" upon accumulating sufficient internal tumor debris.
- **Tumor Debris Uptake:** DCs consume tumor debris present in their surroundings, released upon tumor cell death. The uptake mechanism parallels the “IFNg uptake” approach detailed in the tumors.py.
- **Protein Expression:** Similar to tumor cells, DCs can express PDL1 and MHCI proteins. Activated DCs engage with T cells and, upon activation, gravitate towards lymph nodes to stimulate T cells. For specifics, refer to the lymph_node.py process.

Mechanisms:
1. Death by apoptosis: represents the chance of a dendritic cell undergoing apoptosis uses **`get_probability_step`** method, P, with the parameter “death_apoptosis” for timescale 10 days.
2. Cell Division (for active cells): If the cell is active, a division probability is calculated for the given timestep and division time. If a random number is less than this, the the cell divides.
3. State Transition (for inactive cells): If the cell is inactive and has internal tumor debris count above a certain threshold, it becomes active.
4. Tumor Debris Uptake: The cell takes up tumor debris from the environment up to a limit defined by a rate and the timestep, or until available debris is exhausted.
5. Active Cell Behavior: If the cell is active, it expresses certain proteins at equilibrium levels.

Here are parameters loaded within **`dendritic_cell.py`** and the source of where they are coming from and whether they were varied within the experiments:

Parameter name	Value	Source	Location
Diameter	10 μm	[31]	dendritic_cell.py
Location [x, y]	Vary	Multiplexed Imaging Data	
velocity	3 μm/min	[32]	
death_apoptosis	0.5 (within 4 days)	[33]	
divide_prob	0.5 (within 5 days)	In vitro data	
divide_time	5 days		
internal_tumor_debris_threshold	415,000 (counts)	[34]	
tumor_debris_uptake	300 molecules/cell/hr		
tumor_debris_MW	29,000 g/mol (assuming for HGMB1)	[30]	

REFERENCES

1. Agmon, E., Spangler, R.K., Skalnik, C.J., Poole, W., Peirce, S.M., Morrison, J.H., and Covert, M.W. (2022). Vivarium: an interface and engine for integrative multiscale modeling in computational biology. Bioinformatics *38*, 1972–1979.
2. Bousso, P. (2008). T-cell activation by dendritic cells in the lymph node: lessons from the movies. Nat. Rev. Immunol. *8*, 675–684.
3. Itano, A.A., and Jenkins, M.K. (2003). Antigen presentation to naive CD4 T cells in the lymph node. Nat. Immunol. *4*, 733–739.
4. Hunter, M.C., Teijeira, A., and Halin, C. (2016). T cell trafficking through lymphatic vessels. Front. Immunol. *7*, 613.
5. Liao, K.-L., Bai, X.-F., and Friedman, A. (2014). Mathematical modeling of interleukin-27 induction of anti-tumor T cells response. PLoS One *9*, e91844.
6. Krouglova, T., Vercammen, J., and Engelborghs, Y. (2004). Correct diffusion coefficients of proteins in fluorescence correlation spectroscopy. Application to tubulin oligomers induced by Mg2+ and Paclitaxel. Biophys. J. *87*, 2635–2646.
7. Kurzrock, R., Rosenblum, M.G., Sherwin, S.A., Rios, A., Talpaz, M., Quesada, J.R., and Gutterman, J.U. (1985). Pharmacokinetics, single-dose tolerance, and biological activity of recombinant γ-interferon in cancer patients. Cancer Res. *45*, 2866–2872.
8. Zhao, M., Kiernan, C.H., Stairiker, C.J., Hope, J.L., Leon, L.G., van Meurs, M., Brouwers-Haspels, I., Boers, R., Boers, J., and Gribnau, J. (2020). Rapid in vitro generation of bona fide exhausted CD8+ T cells is accompanied by Tcf7 promotor methylation. PLoS Pathog. *16*, e1008555.
9. Salerno, F., Paolini, N.A., Stark, R., von Lindern, M., and Wolkers, M.C. (2017). Distinct PKC-mediated posttranscriptional events set cytokine production kinetics in CD8+ T cells. Proc. Natl. Acad. Sci. *114*, 9677–9682.
10. Gallegos, A.M., Xiong, H., Leiner, I.M., Sušac, B., Glickman, M.S., Pamer, E.G., and van Heijst, J.W.J. (2016). Control of T cell antigen reactivity via programmed TCR downregulation. Nat. Immunol. *17*, 379–386.
11. Petrovas, C., Price, D.A., Mattapallil, J., Ambrozak, D.R., Geldmacher, C., Cecchinato, V., Vaccari, M., Tryniszewska, E., Gostick, E., and Roederer, M. (2007). SIV-specific CD8+ T cells express high levels of PD1 and cytokines but have impaired proliferative capacity in acute and chronic SIVmac251 infection. Blood, J. Am. Soc. Hematol. *110*, 928–936.
12. Tang, X., Li, Q., Zhu, Y., Zheng, D., Dai, J., Ni, W., Wei, J., Xue, Y., Chen, K., and Hou, W. (2015). The advantages of PD1 activating chimeric receptor (PD1-ACR) engineered lymphocytes for PDL1+ cancer therapy. Am. J. Transl. Res. *7*, 460.
13. Dong, H., Strome, S.E., Salomao, D.R., Tamura, H., Hirano, F., Flies, D.B., Roche, P.C., Lu, J., Zhu, G., and Tamada, K. (2002). Tumor-associated B7-H1 promotes T-cell apoptosis: a potential mechanism of immune evasion. Nat. Med. *8*, 793–800.
14. Zelinskyy, G., Robertson, S.J., Schimmer, S., Messer, R.J., Hasenkrug, K.J., and Dittmer, U. (2005). CD8+ T-cell dysfunction due to cytolytic granule deficiency in persistent Friend retrovirus infection. J. Virol. *79*, 10619–10626.
15. Bouchnita, A., Bocharov, G., Meyerhans, A., and Volpert, V. (2017). Hybrid approach to model the spatial regulation of T cell responses. BMC Immunol. *18*, 1–12.
16. Vodnala, S.K., Eil, R., Kishton, R.J., Sukumar, M., Yamamoto, T.N., Ha, N.-H., Lee, P.-H., Shin, M., Patel, S.J., and Yu, Z. (2019). T cell stemness and dysfunction in tumors are triggered by a common mechanism. Science (80-.). *363*, eaau0135.
17. Boissonnas, A., Fetler, L., Zeelenberg, I.S., Hugues, S., and Amigorena, S. (2007). In vivo imaging of cytotoxic T cell infiltration and elimination of a solid tumor. J. Exp. Med. *204*, 345–356.
18. Mempel, T.R., Henrickson, S.E., and Von Andrian, U.H. (2004). T-cell priming by

dendritic cells in lymph nodes occurs in three distinct phases. Nature *427*, 154–159.
19. Thibaut, R., Bost, P., Milo, I., Cazaux, M., Lemaître, F., Garcia, Z., Amit, I., Breart, B., Cornuot, C., and Schwikowski, B. (2020). Bystander IFN-γ activity promotes widespread and sustained cytokine signaling altering the tumor microenvironment. Nat. Cancer *1*, 302–314.
20. Betts, M.R., and Koup, R.A. (2004). Detection of T-cell degranulation: CD107a and b. Methods Cell Biol. *75*, 497–512.
21. Zhang, M., Park, S.-M., Wang, Y., Shah, R., Liu, N., Murmann, A.E., Wang, C.-R., Peter, M.E., and Ashton-Rickardt, P.G. (2006). Serine protease inhibitor 6 protects cytotoxic T cells from self-inflicted injury by ensuring the integrity of cytotoxic granules. Immunity *24*, 451–461.
22. Böhm, W., Thoma, S., Leithäuser, F., Möller, P., Schirmbeck, R., and Reimann, J. (1998). T cell-mediated, IFN-γ-facilitated rejection of murine B16 melanomas. J. Immunol. *161*, 897–908.
23. Merritt, R.E., Yamada, R.E., Crystal, R.G., and Korst, R.J. (2004). Augmenting major histocompatibility complex class I expression by murine tumors in vivo enhances antitumor immunity induced by an active immunotherapy strategy. J. Thorac. Cardiovasc. Surg. *127*, 355–364.
24. Gong, C., Milberg, O., Wang, B., Vicini, P., Narwal, R., Roskos, L., and Popel, A.S. (2017). A computational multiscale agent-based model for simulating spatio-temporal tumour immune response to PD1 and PDL1 inhibition. J. R. Soc. Interface *14*, 20170320.
25. Eden, E., Geva-Zatorsky, N., Issaeva, I., Cohen, A., Dekel, E., Danon, T., Cohen, L., Mayo, A., and Alon, U. (2011). Proteome half-life dynamics in living human cells. Science (80-.). *331*, 764–768.
26. Celada, A., and Schreiber, R.D. (1987). Internalization and degradation of receptor-bound interferon-gamma by murine macrophages. Demonstration of receptor recycling. J. Immunol. *139*, 147–153.
27. Hoekstra, M.E., Bornes, L., Dijkgraaf, F.E., Philips, D., Pardieck, I.N., Toebes, M., Thommen, D.S., van Rheenen, J., and Schumacher, T.N.M. (2020). Long-distance modulation of bystander tumor cells by CD8+ T-cell-secreted IFN-γ. Nat. Cancer *1*, 291–301.
28. El Darzi, E., Bazzi, S., Daoud, S., Echtay, K.S., and Bahr, G.M. (2017). Differential regulation of surface receptor expression, proliferation, and apoptosis in HaCaT cells stimulated with interferon-γ, interleukin-4, tumor necrosis factor-α, or muramyl dipeptide. Int. J. Immunopathol. Pharmacol. *30*, 130–145.
29. Ersvaer, E., Skavland, J., Ulvestad, E., Gjertsen, B.T., and Bruserud, Ø. (2007). Effects of interferon gamma on native human acute myelogenous leukaemia cells. Cancer Immunol. Immunother. *56*, 13–24.
30. Apetoh, L., Ghiringhelli, F., Tesniere, A., Obeid, M., Ortiz, C., Criollo, A., Mignot, G., Maiuri, M.C., Ullrich, E., and Saulnier, P. (2007). Toll-like receptor 4–dependent contribution of the immune system to anticancer chemotherapy and radiotherapy. Nat. Med. *13*, 1050–1059.
31. Morefield, G.L., Sokolovska, A., Jiang, D., HogenEsch, H., Robinson, J.P., and Hem, S.L. (2005). Role of aluminum-containing adjuvants in antigen internalization by dendritic cells in vitro. Vaccine *23*, 1588–1595.
32. Lämmermann, T., Bader, B.L., Monkley, S.J., Worbs, T., Wedlich-Söldner, R., Hirsch, K., Keller, M., Förster, R., Critchley, D.R., and Fässler, R. (2008). Rapid leukocyte migration by integrin-independent flowing and squeezing. Nature *453*, 51–55.
33. Naik, S.H. (2008). Demystifying the development of dendritic cell subtypes, a little. Immunol. Cell Biol. *86*, 439–452.
34. Yang, D., Chen, Q., Yang, H., Tracey, K.J., Bustin, M., and Oppenheim, J.J. (2007). High

mobility group box-1 protein induces the migration and activation of human dendritic cells and acts as an alarmin. J. Leucoc. Biol. *81*, 59–66.
